# EHMTI-0326. OnabotulinumtoxinA for the treatment of chronic paroxysmal hemicrania: a case report

**DOI:** 10.1186/1129-2377-15-S1-C16

**Published:** 2014-09-18

**Authors:** D Dolezil

**Affiliations:** 1 Prague Headache Center, DADO MEDICAL s.r.o., Prague 2, Czech Republic

## Introduction

Chronic paroxysmal hemicrania (CPH) is characterized by attacks of very severe, unilateral pain, fulfilling criteria of The International Classification of Headache Disorders, 3rd edition, beta version ( ICHD-3 beta) for paroxysmal hemicrania and occurring without a remission period, or with remissions lasting <1 month, for at least 1 year.

## Aims

OnabotulinumtoxinA is now used for the treatment of chronic migraine.We wanted to try the treatment of CPH with OnabotulinumtoxinA, because other treatments have not been effective.

## Patient and methods

We present the case of a patient diagnosed with CPH that lasts for 32 years. Attacks of headache lasted about 15 years without remission. The patient described her headaches as attacks of hemicrania lasted from 15 to 25 minutes with a frequency usually 8 times per day. OnabotulinumtoxinA was infiltrated at eight ipsilateral points. Total dose was 45 units (each intramuscular injection site was 0.1 mL = 5 U onabotulinumtoxinA). Seven points were identical as in the treatment of chronic migraine in the frontal and temporal areas and one point was in infraorbital area.Method applications of onabotulinumtoxinA based on the methodology of application of onabotulinumtoxinA in patients with chronic migraine.

## Results

The CPH showed a dramatic response to onabotulinumtoxinA infiltration. Effect of treatment was evident as early as three weeks after the first injection, gradually improved, it takes seven months from start of treatment with 3-monthly infiltrations.

## Conclusions

The treatment of CPH with OnabotulinumtoxinA significantly improved the quality of life of the patient.

